# Estimating the prevalence of exclusive breastfeeding with data from household surveys: Measurement issues and options

**DOI:** 10.3389/fnut.2023.1058134

**Published:** 2023-03-24

**Authors:** Thomas W. Pullum, Karleen Gribble, Seema Mihrshahi, Bindi Borg

**Affiliations:** ^1^ICF International, The Demographic and Health Surveys Program, Rockville, MD, United States; ^2^School of Nursing and Midwifery, Western Sydney University, Penrith, NSW, Australia; ^3^Department of Health Sciences, Faculty of Medicine and Health Sciences, Macquarie University, Sydney, NSW, Australia

**Keywords:** exclusive breastfeeding, infant feeding, IYCF indicators, demographic and health survey data, WHO EBF recommendation

## Abstract

The importance of breastfeeding for infant and maternal health is well established. The World Health Organization recommends that all infants be exclusively breastfed until they reach 6 months of age. The standard indicator to measure adherence to this criterion is the percentage of children aged 0–5 months who are currently being exclusively breastfed. This paper proposes supplementary measures that are easily calculated with existing survey data. First, for an accurate assessment of the WHO recommendation, we estimate the percentage of infants who are being exclusively breastfed at the exact age of 6 months. Second, an adjustment is proposed for prelacteal feeding. These two modifications, separately and in combination, are applied to data from 31 low-and middle-income countries that have participated in the Demographic and Health Surveys Program since 2015. There is considerable variation in the effects across countries. The modifications use existing data to provide a more accurate estimate than the standard indicator of the achievement of the exclusive breastfeeding until 6 months recommendation.

## Introduction

Breastfeeding is the biologically normal way of feeding human infants. It provides protection from infectious disease (1–3), facilitates normal development and cognition, reduces risk of chronic diseases and is also beneficial for the mother (3–5). It is especially crucial in low-and middle-income countries where it is estimated that over 800,000 child deaths or (11.6% of all deaths) are associated with infants not being breastfed as recommended (3).

The mechanisms by which breastfeeding has a protective effect on infant health, while other forms of feeding have a deleterious impact, are still being studied. They include immune protection conferred by breastmilk in the form of antibodies and white cells to resist infection, glycans that bind to pathogens, and breastmilk’s oligosaccharides and lactose which nurture the growth of beneficial bacteria (6). Breastmilk consumption has a demonstrated long-term positive effect on the gut microbiome of infants which in turn has been shown to reduce intestinal and other morbidities, and enables positive neurodevelopmental outcomes (7). On the other hand, feeding foods or liquids other than breastmilk, including infant formula, deprive infants of breastmilk’s protection while introducing pathogens, as well as potentially damaging the intestinal lining, and increasing susceptibility to infection. Even small amounts of other foods or liquid feeds can be damaging (6).

As a global public health recommendation, the World Health Organization (WHO) and the United Nations Children’s Fund (UNICEF) advised in the Global Strategy for Infant and Young Child Feeding that “infants should be exclusively breastfed for the first 6 months of life to achieve optimal growth, development and health” (8). The Global Strategy explicitly recommends exclusive breastfeeding over any breastfeeding because feeding other foods or liquids to infants under 6 months increases their risk of infectious disease. The World Health Assembly has set a global target of 50% of children exclusively breastfeeding in the first 6 months of life by 2025 (9).

To ensure these targets are reached, reliable and accurate monitoring of exclusive breastfeeding (EBF) rates is critical. This is especially the case in low-and middle-income countries (LMICs) where infant mortality rates may be high (9). Household surveys are the main source of information about breastfeeding in LMICs. The largest survey programs are the Demographic and Health Surveys (DHS) and the Multiple Indicator Cluster Surveys (MICS), sponsored by the United States Agency for International Development (USAID) and UNICEF, respectively. The DHS and MICS are virtually identical in terms of sampling designs, questionnaires, indicators, and reporting. This paper refers to DHS surveys, but its findings can be applied to other household surveys that collect breastfeeding data, including MICS surveys. In both programs, estimates are derived from mothers’ reports of breastfeeding and consumption of liquids and foods by the child in the 24 h before the survey^1^.

The large-scale measurement and reporting of EBF prevalence is complex and necessarily imperfect (10, 11). However, for countries to create policy and implement programming to improve exclusive breastfeeding rates, it is essential to have the most accurate estimates possible.

This paper focuses on the measurement and reporting of prevalence estimates of EBF. First, it highlights a discrepancy between the EBF recommendation and the standard indicator that is used to monitor it (12). The WHO recommendation is that “infants should be exclusively breastfed for the first 6 months of life” (8). However, the DHS and other indicators measure EBF as the percentage of children less than 6 months of age who are currently exclusively breastfed, that is, the prevalence of EBF at age 0–5 completed months. The prevalence of EBF declines steadily with the age of the child, so the prevalence in the interval 0–5 months is necessarily greater than the prevalence at the exact age of 6 months. Thus, the standard indicator overestimates the percentage of children meeting the EBF recommendation and is frequently misinterpreted (11). This gives a falsely positive view of EBF prevalence and undermines the urgency of promoting EBF.

Second, the paper describes how prelacteal feeding affects EBF prevalence. This refers to any liquids and foods that are given to newborns before the first breastfeed, usually on the first day of life (13). The DHS’s working definition of prelacteal feeding is anything other than breastmilk given within the first three^2^ days after birth (14). Prelacteal feeding greatly increases the risk of illness in infants and young children (15, 16), by disrupting the microbiome, damaging the intestinal lining, and introducing pathogens (17) thereby increasing infant morbidity and mortality. Nevertheless, prelacteal feeding is highly prevalent, particularly in LMICs (16). Prelacteal feeds and delayed initiation of breastfeeding are strongly associated, and with increased mortality rates in infants (18). However, the standard indicator for EBF does not account for prelacteal feeds, thereby resulting in an overestimate of EBF.

This paper describes alternative calculations of the prevalence of EBF, with the aim of clarifying the standard indicator and describing additional options for reporting EBF prevalence. We use the data from 31 DHS surveys conducted since 2015 that included the relevant questions about breastfeeding and for which data files were available from the DHS website^3^ by 31 August 2022. For countries that had more than one DHS survey within that interval, we use only the most recent survey.

## Methodology

Five concepts are crucial for interpreting estimates of EBF prevalence: current (breastfeeding) status, age, exact age, prevalence of EBF in an age interval, and prevalence of EBF at an exact age.

### Current status

Mothers can usually readily say whether a child was ever breastfed or is still breastfeeding (19). However, a mother cannot respond reliably to a question about whether the child was exclusively breastfed from birth until a specific age or time in the past (20). Therefore, the DHS assesses whether the child is exclusively breastfed at the time of the survey with “current status” referring to the 24 h before the interview. Current feeding status is determined in the DHS through multiple questions on whether the child was ever breastfed, and their consumption or not of liquids and foods other than breastmilk in the past 24h The variable that is constructed from the DHS data files has been labeled “ebf.” It takes the value ebf = 1 (yes) if the child consumed only breastmilk during the 24 h prior to the interview, or ebf = 0 (no) if the child consumed other liquids or foods in that time interval^4^.

However, ebf = 1 does not ensure that the child *never* consumed anything other than breastmilk. While it is recommended that EBF be continuous from birth to the introduction of other liquids and foods at 6 months, it may, in fact, be subject to interruptions. These could include prelacteal feeding, subsequent feeding of liquids and foods other than breastmilk prior to the past 24 h and feeding of liquids and foods other than breastmilk by other caregivers without the mother’s knowledge. Measurement error is also possible in the classification into ebf = 0 and ebf = 1 for the last 24 h and interpretation error occurs if the classification is understood as indicating as continuous EBF since birth.

### Age and exact age

The distinction between “age” and “exact age” is important for the measurement of EBF. Exact age is calculated from the elapsed number of days between the reported date of the birth and the date of the interview with the mother. For DHS, “exact age 6 months” refers to exactly 183 days after birth. Age, by contrast, as the term is normally used, refers to an interval. For example, age 0–5 months is a six-month interval extending from birth up to, but not including exact age of 6 months.

### Prevalence of EBF in an age interval, and prevalence of EBF at an exact age

The “prevalence of EBF at age 0–5 months” is the number of children in the age interval who are exclusively breastfed during the previous 24 h (the numerator) divided by the total number of children in the age interval (the denominator) times 100. This is what DHS currently calculates and reports. The WHO recommends that children be exclusively breastfed for 6 months, implicitly setting a target of 100% prevalence of EBF at exact age 6 months. This paper argues that the prevalence of 24-h recall of EBF at exact ages can be calculated and reported in much the same way that the prevalence of EBF is estimated for age intervals.

**Figure 1:**
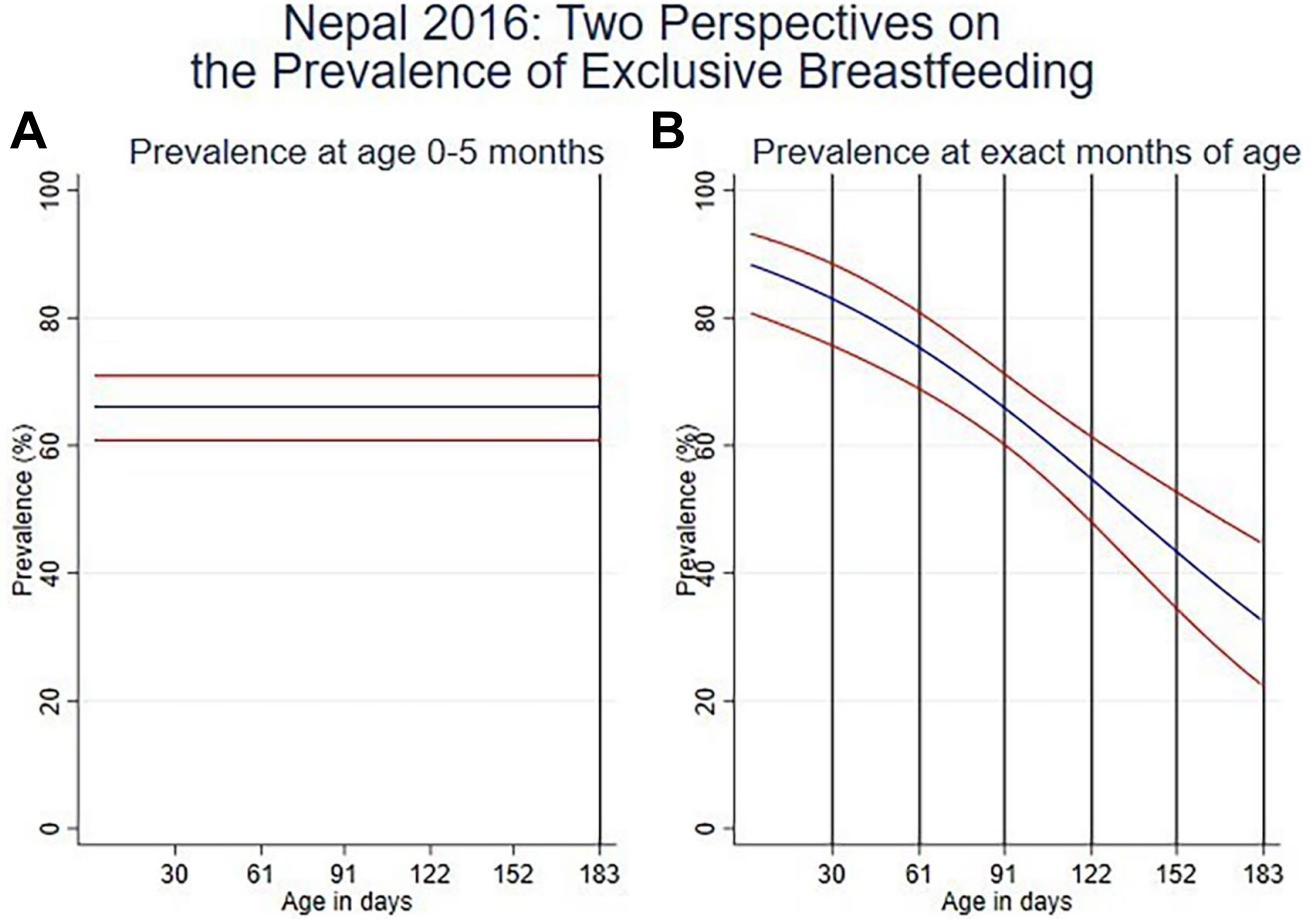
**(A)** Prevalence of EBF in the age interval 0–5 months and **(B)**. Prevalence at exact ages in months.

Data from the Nepal 2016 DHS survey (Figure 1) illustrates the difference between estimates based on the two interpretations of the child’s age. Nepal was chosen as an example of a country with a high prevalence of EBF at age 0–5 months, but a much lower prevalence of EBF at exactly 6 months. Figure 1 contains two panels; in each, the vertical axis shows 24-h EBF prevalence, and the horizontal axis shows age in days from birth to 6 months (rounded to the nearest day). The EBF estimate is shown by the blue line and upper and lower bounds of the 95% confidence interval are shown by the red lines.

Figure 1A, the panel on the left, illustrates use of ‘age’ as the variable and shows an EBF prevalence of 66.1% for ages 0–5 months, as indicated in the Nepal DHS report (21). Figure 1B, the panel on the right, illustrates the alternative perspective on prevalence where ‘exact age’ is used and shows that EBF prevalence is highest in the first 2 months, declining to 65.8% (CI 95%: 60.1–71.2%) at 3 months and 32.5% (CI 95%: 22.4–44.6%) at 6 months.

Prevalence of EBF takes only two values (coded ebf = 0 and ebf = 1) and is analyzed with logit regression, which adjusts for the survey design (including sample weights, clusters, and stratification) and produces confidence intervals as well as point estimates. The usual estimate of EBF prevalence, corresponding with Figure 1A on the left, is descriptive and uses logit regression to smooth the month-to-month data for children aged 0–5 months with no covariates. The only modification required for specific values of age, as in Figure 1B on the right, is that age in days is included as a linear covariate. Linearity on the logit scale produces a familiar logistic shape on the scale of the prevalence. More details on this are provided in Supplementary File S1.

In addition, the DHS collects data on prelacteal feeding, with a question which asks whether the child was given anything to drink other than breastmilk in the first 3 days after delivery. This demonstrates an important limitation of using 24-h EBF recall alone as a proxy for EBF since birth.

EBF prevalence can be recalculated to take prelacteal feeding into account by assigning ebf = 0 to children for age 0–5 months who received prelacteal feeding.

## Results

This paper presents analyses of the DHS data for 31 countries of prevalence of exclusive breastfeeding in the age interval 0–5 months; EBF at the exact age of 6 months; EBF 0–5 months with children who received prelacteal feeds excluded from the EBF estimate; and EBF at 6 months with children who received prelacteal feeds excluded from the EBF estimate.

Table 1 lists the DHS countries from lowest to highest EBF 0–5 months and provides the numerical values (other than confidence intervals) for the figures below. It also includes the unweighted and weighted number of children used for each estimate. The number of children reflects the sample size and the fertility level in the country. It ranges from 177 in Armenia to 1,636 in Malawi, except for the much larger frequencies of 3,193 in Nigeria and 22,865 in India.

**Table 1:** Estimates of EBF, showing the effects of changing the standard calculation and reporting to one that corresponds with the WHO criterion for age, and adjusting for prelacteal feeding (31 DH surveys conducted since 2015).

A	B	C	D	E	F	G	H	I	J
Survey and date	EBF 0-5 m	EBF at 6 m	Difference (C-B) (EBF 6 m–EBF 0-5 m)	EBF 0-5 m minus prelacteal feeding	Difference (E-B) (EBF 0-5 m minus prelacteal feeding–EBF 0-5 m)	EBF at 6 m minus prelacteal feeding	Difference (G-C) (EBF at 6 m minus prelacteal feeding–EBF at 6 m)	n (unweighted)	n (weighted)
	%	%	%	%	%	%	%		
Albania 2017	36.7	22	−14.8	31.4	−5.3	25.2	−11.5	285	283.7
Angola 2015–16	37.7	10.8	−26.8	36.1	−1.5	10.9	−26.8	1,620	1486.2
Armenia 2015–16	44.5	8	−36.4	42.5	−2	8.4	−36.1	177	172
Bangladesh 2017–18	65	25.9	−39.1	49	−15.9	24	−41	971	953.5
Benin 2017–18	41.7	12	−29.7	37.8	−3.9	11.8	−29.9	1,381	1386.6
Cameroon 2018	40.2	24.5	−15.7	32.7	−7.4	17.6	−22.6	986	1030.2
Ethiopia 2016	57.5	29.5	−28	55.7	−1.7	28.8	−28.7	1,092	1184.9
Gambia 2019–20	53.6	19	−34.6	48.9	−4.7	18.2	−35.4	1,003	896.6
Guinea 2018	33.4	13.2	−20.3	26.6	−6.8	11.2	−22.2	916	912.5
Haiti 2016–17	39.9	7.9	−32	36.2	−3.6	7.9	−31.9	700	660.3
India 2019–21	63.8	42.2	−21.6	55.7	−8.1	38.2	−25.6	22,865	22404.4
Jordan 2017–18	25.6	6.4	−19.2	17.6	−8	4.8	−20.8	1,218	1059.4
Liberia 2019–20	55.2	28.9	−26.3	53.5	−1.7	27.8	−27.5	562	554.8
Madagascar 2021	55.2	16.6	−38.6	44.4	−10.8	16.8	−38.4	1,291	1260.6
Malawi 2015–16	61.2	24.7	−36.4	59.2	−1.9	25.1	−36.1	1,636	1627.4
Maldives 2016–17	63.5	47	−16.5	59.9	−3.6	47.2	−16.3	288	280.8
Mali 2018	40.5	15.2	−25.3	34.5	−6	12.2	−28.3	997	1,087
Mauritania 2019–21	41.1	18.1	−23	34.4	−6.7	13.9	−27.2	1,202	1,250
Nepal 2016	66.1	32.5	−33.6	48.8	−17.2	24	−42.1	467	443.2
Nigeria 2018	28.8	15.6	−13.3	23	−5.9	12.5	−16.3	3,193	3218.7
Pakistan 2017–18	47.5	32.6	−15	15.4	−32.1	9.4	−38.1	1,117	1138.9
Papua New Guinea 2016–18	62.3	25.8	−36.5	56.2	−6.1	20.8	−41.6	886	903
Rwanda 2019–20	80.9	62.5	−18.4	78.8	−2.1	61.8	−19.1	747	781.5
Sierra Leone 2019	54.2	18.9	−35.3	51.4	−2.7	17.4	−36.8	994	968.7
South Africa 2016	31.6	15.8	−15.8	27.1	−4.5	13.7	−17.9	346	345.3
Tajikistan 2017	36.2	10.2	−25.9	31.7	−4.5	9.3	−26.9	553	587.8
Tanzania 2015–16	59.2	14.6	−44.6	52.2	−7	16.9	−42.3	1,015	998.5
Timor-Leste 2016	51.2	29	−22.1	43.9	−7.3	28.2	−23	743	728
Uganda 2016	65.7	32.8	−32.9	51.7	−14.1	31.8	−34	1,482	1443.3
Zambia 2018	70	26.1	−43.9	65.4	−4.5	28.1	−41.9	1,019	1019.7
Zimbabwe 2015	48	11.1	−36.9	45	−3	11.6	−36.3	603	612.1

Column B shows exclusive breastfeeding prevalence from 0 to 5 months. This is the estimate that is reported in the DHS. Column G shows the prevalence of exclusive breastfeeding at six completed months of age excluding infants who received prelacteal feeds. This is a more accurate estimate of infants who of the proportion of children who are exclusively breastfed until 6 months of age, as recommended by WHO.

It is worth noting that there are significant variations in the differences between EBF 0-5 m and EBF 6 m, ranging from differences of 13 to 45 percentage points. When prelacteal feeding is taken into account, the difference is even larger. Furthermore, 15 out the 31 countries have not reached the target of 50% EBF 0-5 m. (Table 1 gives the numerical values of the estimates that are shown in Figures 4, 5, and 6, along with the differences from the standard estimates for months 0–5).

**Figure 2:**
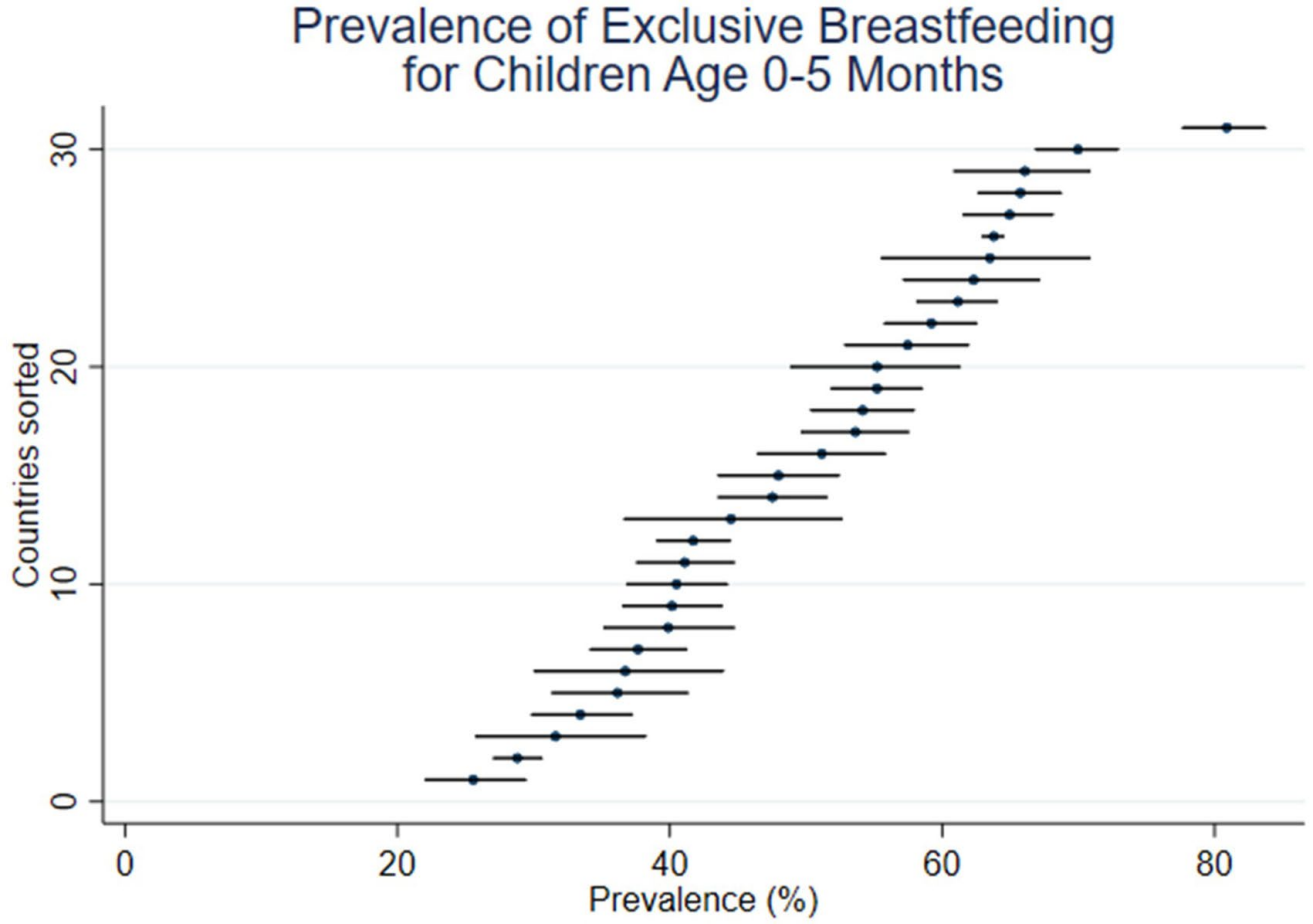
Prevalence of EBF in age interval 0–5 months (31 DHS surveys conducted since 2015).

**Figure 3:**
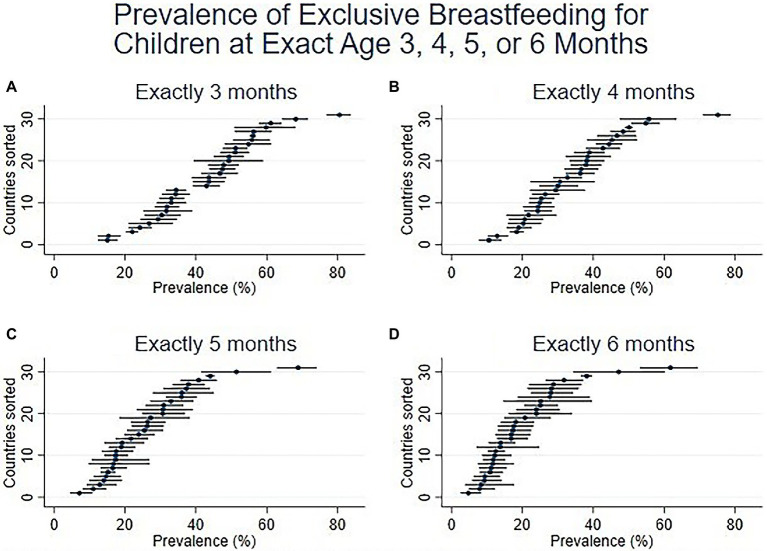
Prevalence of EBF at exact ages in months, namely **(A)**. Exactly 3 months, **(B)**. Exactly 4 months, **(C)**. Exactly 5 months, and **(D)**. Exactly 6 months (31 DHS surveys conducted since 2015).

**Figure 4:**
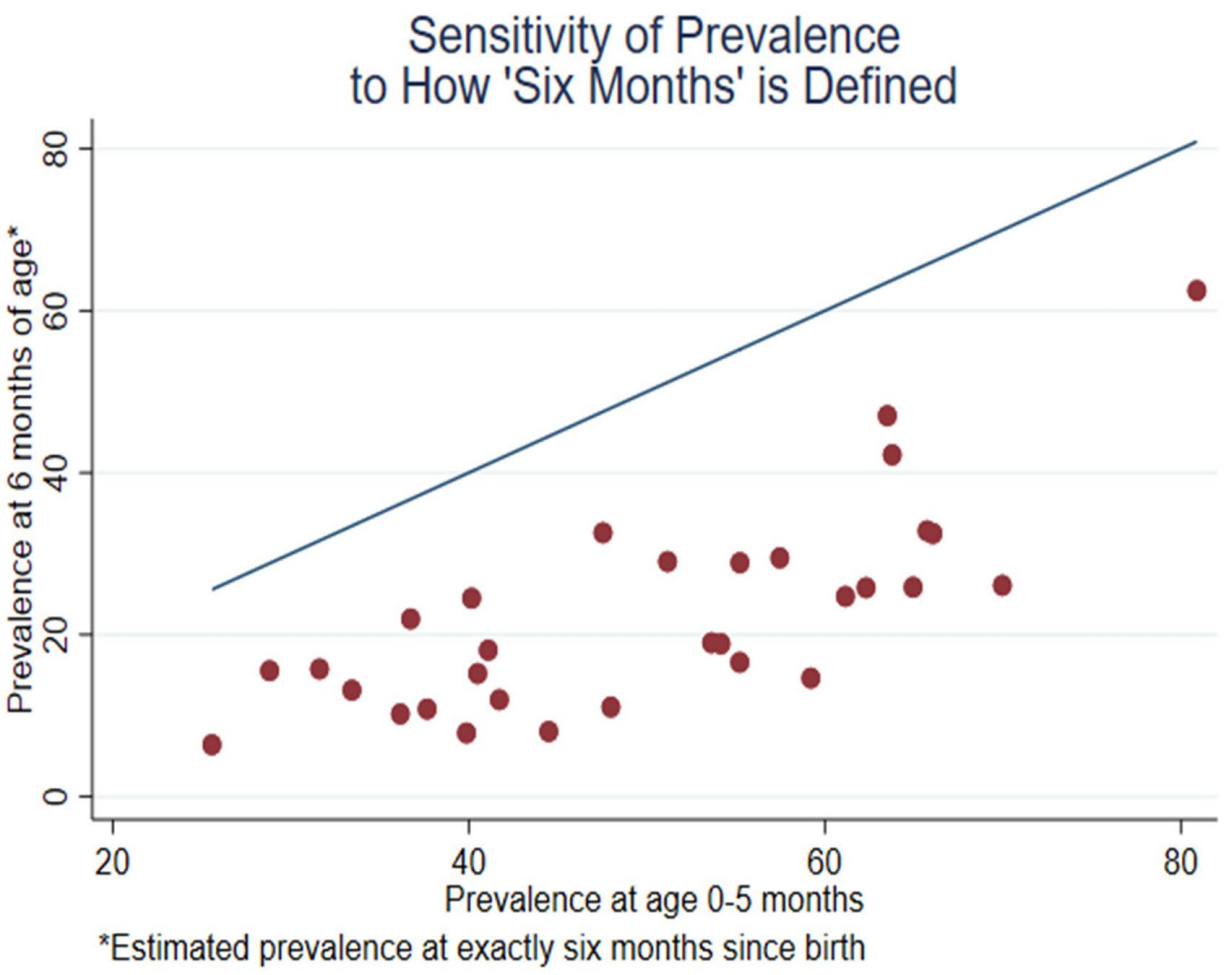
Prevalence of EBF at exact age 6 months compared with the unadjusted prevalence at age 0–5 months (31 DHS surveys conducted since 2015).

**Figure 5:**
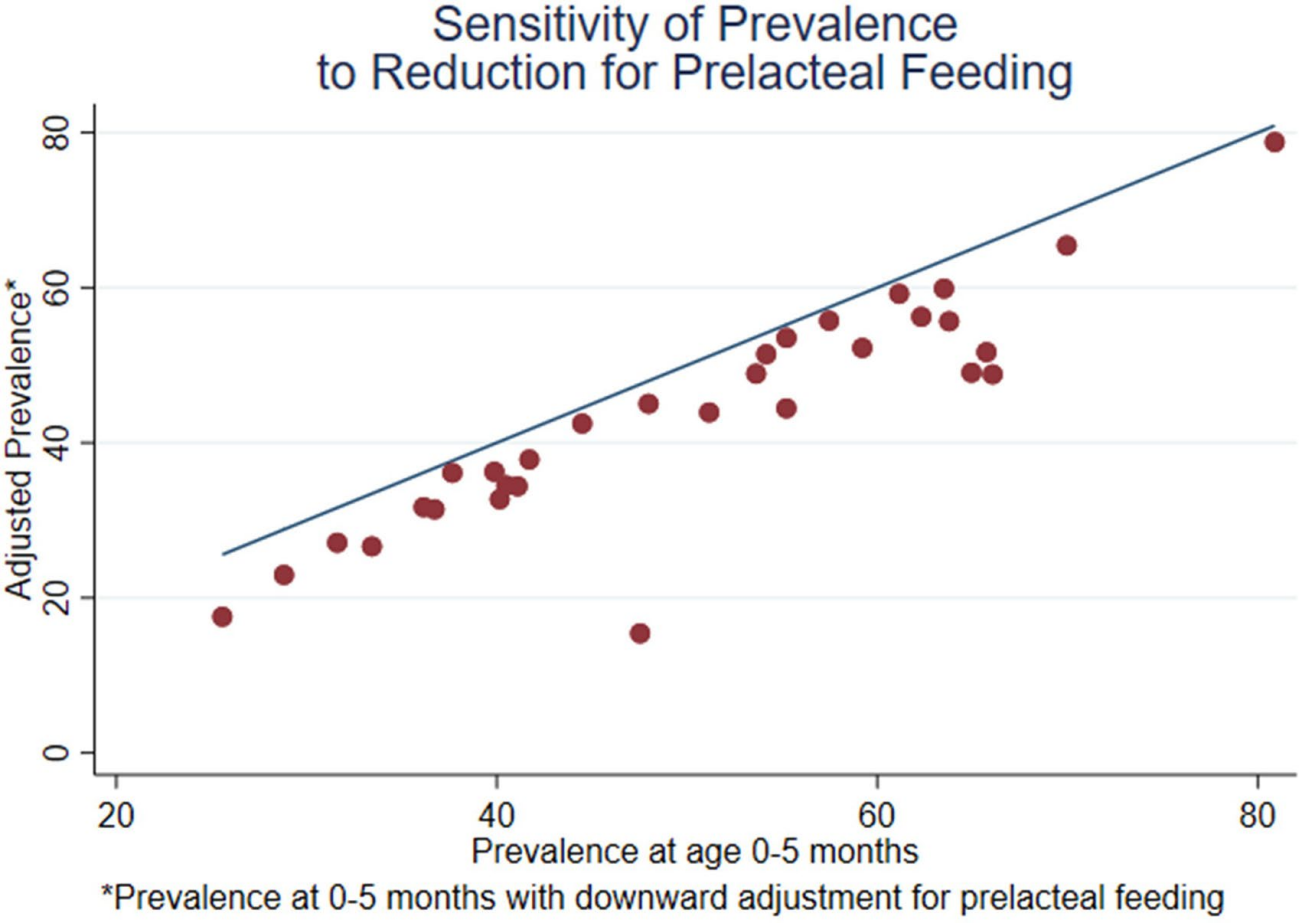
Prevalence of EBF at age 0–5 months, adjusted for prelacteal feeding, compared with the unadjusted prevalence at age 0–5 months (31 DHS surveys conducted since 2015).

The results of the analyses showing the differences between alternative approaches to measuring the prevalence of EBF in the 31 surveys are shown in Figures 2–5.

**Figure 6:**
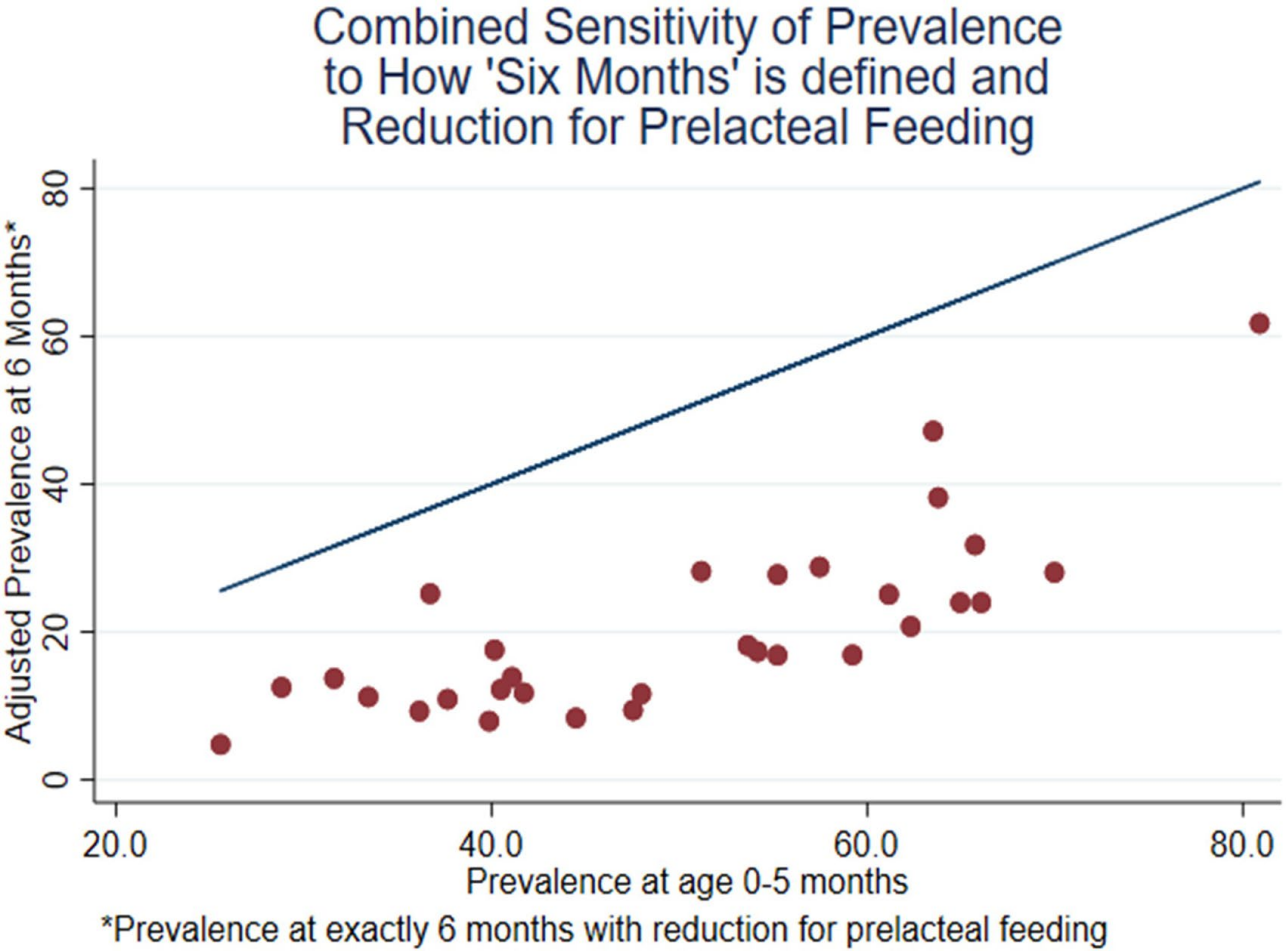
Prevalence of EBF at exact age 6 months, adjusted for prelacteal feeding, compared with the unadjusted prevalence at age 0–5 months (31 DHS surveys conducted since 2015).

Figures 2 and 3 present estimates using the two perspectives on age in five graphs. Prevalence is shown on the horizonal axis, with the countries sorted from lowest to highest point estimates. For each country, a short horizontal line shows the 95% confidence interval, with a dot for the point estimate. Figure 2 (like Figure 1A) shows the prevalence of EBF, as usually presented, in the age interval 0–5 months, ranging from the lowest levels in Jordan (25.6%) and Nigeria (28.8%) to the highest levels in Zambia (70.0%) and Rwanda (80.9%).

Figure 3 includes four panels, showing the prevalence and confidence intervals at exact ages of 3, 4, 5, and 6 months. The four panels all have the same vertical and horizontal axes as Figure 2. The countries are sorted by prevalence at 6 months (this is not necessarily the same as the sorted order by 0-5 months in Figure 2). The panels in Figure 3 show the steady reduction in EBF prevalence as children get older. Of note, it also shows a gradual reduction, from month to month, in the range of prevalence across countries. By 6 months, the EBF prevalence ranges from the lowest levels in Jordan (6.4%), Haiti (7.9%), and Armenia (8.0%) to the highest levels in India (42.2%) Maldives (47.0%), and Rwanda (62.5%). Figure 3d, the bottom right panel (prevalence at exactly 6 months), is a more accurate depiction than Figure 2 of the achievement of the WHO recommendation that all children be exclusively breastfed for the first six months of life.

Figure 4 is a scatterplot with a red dot for each survey, comparing the estimated prevalence at exactly 6 months (the vertical axis) with the estimated prevalence at 0–5 months (the horizontal axis). The figure includes a straight line representing equality of the two measures; if there were no difference between the two estimates, the red dots would be on the line. The vertical distance between the points and the line is the difference between the two measures. This difference ranges from −13.3% for Nigeria to −44.6% for Tanzania. The difference tends to be greater if the prevalence at 0–5 months is greater. The outlier on the right is Rwanda, which has the highest levels on both axes (also seen in Figures 2 and 3). The difference, −18.4%, is relatively small, compared with the other surveys.

Figure 5 is similar to Figure 4 but compares the adjusted prevalence of EBF, after re-classifying the children who received prelacteal feeding, to the unadjusted prevalence at 0–5 months. For each country, the difference is the vertical distance between the blue line and the red dot for that country. For both axes, the reference age is the interval 0–5 months. For most surveys, the difference is small. However, the difference is 8.0% or more in 7 surveys: Pakistan (−32.1%), Nepal (−17.2%), Bangladesh (−15.9%), Uganda (−14.1%), Madagascar (−10.8%), India (−8.1%), and Jordan (−8.0%). This list includes all the surveys that are in South Asia, but it is not limited to South Asia; the effect is greater in Uganda and Madagascar than in India. The outlier at the bottom of the figure represents Pakistan. The effect is about twice as large in Pakistan as in Nepal or Bangladesh.

The combined effect of the two adjustments is shown in Figure 6. The horizontal axis is the usual estimate of EBF prevalence for months 0–5. The vertical axis is the estimate for exact age 6 months, with removal of children who had prelacteal feeding shifted from ebf=1 to ebf=0. The vertical distance between the line and the point is the net change in the estimate due to the combined adjustment. The two adjustments are usually additive but, in some countries, they offset each other. For example, Pakistan is not an outlier in Figure 6, because its relatively high level of sustained breastfeeding tends to offset its high level of prelacteal feeding.

## Limitations

The principal limitation of this study is the potential for recall bias. Highly accurate measurements of exclusive breastfeeding from birth to 6 months can only be carried out prospectively, given the limitations of maternal recall (20). The data for this analysis are based on recall of the three-day interval immediately following the birth (for prelacteal feeding) and on the past 24 h (for exclusive breastfeeding). One study suggested that recall bias in relation to prelacteal feeding might be minimal (16), and where it exists, there is little reason to suspect that there would be differential recall that would affect results (22). Twenty-four hour recall is practical and has low recall bias, but tends to overestimate exclusive breastfeeding (23, 24). However, a mother’s recall of intake could still be incorrect, and health workers or other household members may have given the child other liquids or foods without the mother’s knowledge. By taking the data at face value, we produce estimates of the duration and prevalence of exclusive breastfeeding that are necessarily biased upwards. Despite the limitations of maternal recall of the first few days following birth and over the past 24-h (25, 26), these measures continue to be used in most large surveys such as the DHS. Thus, the majority of studies that report on exclusive breastfeeding suffer the same limitation. Hence, our estimation of breastfeeding at 6 months of age still gives a more accurate estimate than the standard indicator of the achievement of the recommendation of exclusive breastfeeding until 6 months.

A second limitation is that the procedure to estimate the prevalence of exclusive breastfeeding at exactly 6 months is based on a simplifying assumption that the trajectory during the first 6 months is linear on the logit scale. It is possible that the trajectory is not so simple, but the current status data do not provide sufficient statistical power for fitting a more complex pattern. Again, despite this limitation, our estimation is a more accurate indicator of exclusive breastfeeding until 6 months.

## Discussion and conclusions

Prevalence of EBF at 6 months is necessarily less than the prevalence at 0–5 months. The estimates would only be the same if all children were exclusively breastfed for 6 months, in which case, both would be 100%. Exclusive breastfeeding status in Rwanda comes closest to this, but even there, almost 40% of infants are not exclusively breastfed at 6 months of age. Many other countries show even larger discrepancies when estimating EBF using the alternative methods.

This paper demonstrates the limitations to the standard EBF calculation for the range 0–5 months and shows that a calculation of EBF at exact age of 6 months, and taking prelacteal feeding into account, provides a more accurate assessment of infant feeding practices. EBF prevalence at exactly 6 months is a more accurate indication of how well countries are progressing toward the achievement of the WHO recommendation that infants be exclusively breastfed for 6 months. In addition, taking into account data collected on prelacteal feeding in the DHS would also assist in providing a more accurate assessment of the state of infant feeding practice, particularly for countries where prelacteal feeds are common. For example, the Nepal DHS 2016 reports a prevalence of 66% EBF 0–5 months. However, the actual prevalence of children who are exclusively breastfed, including not receiving prelacteal feeding, until 6 months of age is 24%. Moreover, only one country in the dataset, Rwanda, is even halfway toward meeting the recommendation. It is unlikely that many countries will even reach the much more modest target of 50% EBF in the first 6 months.

This more accurate assessment of whether infants are being fed in accordance with WHO recommendations is essential if adequate resources are to be committed to promoting and protecting breastfeeding, including exclusive breastfeeding.

Based on the analysis of 31 DHS surveys conducted since 2015, we suggest that future monitoring of EBF include two indicators to supplement the estimated prevalence of EBF for children 0–5 months which is currently in use. The additional indicators are the estimated prevalence of EBF at exact age 6 months with a deduction for the prevalence of prelacteal feeding.

The methods suggested in this paper utilize existing DHS questions and data and do not require any additional questions or increase the burden on respondents. The statistical analysis involved in calculating EBF prevalence at exact age and taking prelacteal feeding into account is not onerous.

The suggested modifications will not provide a perfect assessment of EBF. Using 24-h recall has limitations, and more accurate measures of EBF using prospective research are difficult and expensive. However, in order to have sound policy and programming to contribute to the goal of exclusive breastfeeding for all infants for their first 6 months, it behooves us to have the most accurate estimates possible using the data that we already have. The recommendations in this paper would make a significant contribution toward achieving that goal.

## Data availability statement

Publicly available datasets were analyzed in this study. This data can be found here: https://dhsprogram.com/

## Author contributions

TP and BB conceptualized the study, TP conducted the statistical analysis and drafted the initial version of the manuscript. KG, SM, and BB provided significant additional input. All authors and approved the final manuscript.

## Conflict of interest

The authors declare that the research was conducted in the absence of any commercial or financial relationships that could be construed as a potential conflict of interest.

## Publisher’s note

All claims expressed in this article are solely those of the authors and do not necessarily represent those of their affiliated organizations, or those of the publisher, the editors and the reviewers. Any product that may be evaluated in this article, or claim that may be made by its manufacturer, is not guaranteed or endorsed by the publisher.

## Supplementary material

The Supplementary material for this article can be found online at: https://www.frontiersin.org/articles/10.3389/fnut.2023.1058134/full#supplementary-material

## Footnotes

^1^Questions about breastfeeding are only asked about the youngest child under 2 years of age who is living with the mother, and modifications are made if the child was part of a multiple birth.

^2^Surveys in this dataset define prelacteal feeding as anything other than breastmilk given within the first 3 days after delivery. However, future DHS surveys (DHS 8 Model Questionnaire Phase 8) will define prelacteal feeding as anything other than breastmilk given within the first 2 days after delivery. Countries may choose to keep the 3 day definition for continuity in surveys.

^3^ www.dhsprogram.com

^4^"EBF," with upper case letters, refers to the general concept of exclusive breastfeeding. "ebf," with lower case letters, refers to the specific binary outcome defined here.
