# Supplementary material for: Estimating the prevalence of exclusive breastfeeding with data from household surveys: Measurement issues and options
Source: Front Nutr. 2023 Mar 24;10:1058134. doi: 10.3389/fnut.2023.1058134 (PMC10080117; doi:10.3389/fnut.2023.1058134)
Supplement: Supplementary file 1 [file Data_Sheet_1.docx]

**Supplementary file 1**

In order to enable replication of the estimates, Supplementary file 1 provides precise definitions and details on analytical procedures.

The women in DHS surveys are age 15-49 and *de facto* residents^[[1]](#footnote-1)^ of the sampled households. Women—and their children—are omitted if they are outside the age range 15-49 or outside the household population. To be included, a child must be under two years of age AND be living with the mother AND be the youngest such child. These are the children about whom dietary information is obtained to determine whether ebf=0 or ebf=1. If a child included under these criteria is part of a multiple birth, then any other children in that multiple birth who are alive and living with the mother will be included and assigned the same value of ebf. Otherwise, children other than the youngest child are omitted and children who are not living with the mother are omitted. Children who are in a sampled household but not living with their mother, because she is living elsewhere or has died, are omitted. The number of living children under 6 months of age omitted from the denominator for these reasons is usually very small. There is little basis for an assumption about whether those children should be classified with ebf=0 or ebf=1, which would be required for them to be added into the denominator.

The prevalence is the percentage of children, in an age interval or at an exact age, who have ebf=1. As with all indicators in the final reports, the percentage for age interval 0-5 completed months of age is routinely calculated by DHS using CSPro. It is approached entirely from the perspective of data processing: for children in the age interval, the weighted number of cases with ebf=1 is divided by the weighted number of cases with ebf=0 or 1, and the ratio is multiplied by 100. The standard table that includes this indicator provides the weighted number of cases on which the estimate is based, but the estimate is not accompanied by a standard error or a confidence interval. DHS reports include an appendix that provides standard errors for many indicators, but not this one.

The prevalence of an outcome such as ebf, which takes only two values that can be coded 0 and 1, is easily analysed with logit regression using the microdata file for children. It can be shown both empirically and mathematically that the estimate of prevalence in an age interval using logit regression matches exactly with the conventional estimate. The matching logit regression has ebf as the outcome and no covariates. A simplified set of commands is given below. In this code, r(table) is a saved matrix of results from the logit regression. That matrix includes the point estimate and the lower and upper ends of a 95% confidence interval for the only parameter in the model, the intercept. Those numbers are extracted from the matrix as scalars and then converted to fitted proportions with the inverse logit transformation. The proportions are multiplied by 100 to get percentages.

svyset v001 [pweight=v005], strata(v023) singleunit(centered)

svy: logit ebf if age_in_months<6

matrix T=r(table)

scalar sP=T[1,1]

scalar sL=T[5,1]

scalar sU=T[6,1]

scalar sprev_0to5_sP=100*exp(sP)/(1+exp(sP))

scalar sprev_0to5_sL=100*exp(sL)/(1+exp(sL))

scalar sprev_0to5_sU=100*exp(sU)/(1+exp(sU))

The estimate of prevalence at specific exact ages is obtained from a logit regression that is identical except that it includes “age_in_days” as a covariate. After the estimation command, the predict command is used to produce fitted values “yhat” on the logit scale and standard errors of the prediction “sd” (also on the logit scale, by default). The fitted logits have a normal sampling distribution. The last three lines produce a fitted line (“yhat_P”) and 95% confidence bands (“yhat_L” and “yhat_U”) on the prevalence scale, again with the inverse logit transformation and a factor of 100. There are then various ways to extract the values of these three functions at exact days. The procedure used for this paper calculates completed months of age as multiples of (365.25/12)=30.4375 days and then rounds those numbers to the nearest hundredth.

svyset v001 [pweight=v005], strata(v023) singleunit(centered)

svy: logit ebf age_in_days if age_in_months<6

predict yhat_xb,xb

predict sd,stdp

gen yhat_P=100*exp(yhat_xb)/(1+exp(yhat_xb))

gen yhat_L=100*exp(yhat_xb-1.96*sd)/(1+exp(yhat_xb-1.96*sd))

gen yhat_U=100*exp(yhat_xb+1.96*sd)/(1+exp(yhat_xb+1.96*sd))

The median duration is obtained as the value of “age_in_days” for which yhat_P=.5. This value is converted to months by dividing by 30.4375 and rounding to the nearest hundredth.

Logit regression is not the only statistical method that could be used to produce these estimates. Other possibilities include regression with splines, lowess regression, and hazard models. Alternative methods would produce somewhat different estimates.

1. These women slept in the household the previous night but in some cases are not usual (*de jure*) residents. [↑](#footnote-ref-1)
